# Occupancy Statistics and Entropy in Bose Systems

**DOI:** 10.3390/e28080911

**Published:** 2026-08-13

**Authors:** Arnaldo Spalvieri

**Affiliations:** Dipartimento di Elettronica, Informazione e Bioingegneria, Politecnico di Milano, 20133 Milano, Italy; arnaldo.spalvieri@polimi.it

**Keywords:** thermodynamics, canonical systems, occupancy numbers, Bose–Einstein ideal quantum gas, grand canonical distribution, canonical distribution, multinomial distribution

## Abstract

In this work, we compare three formulations of thermodynamic entropy for a non-interacting bosonic gas: (i) the grand-canonical Bose–Einstein entropy, (ii) the finite-N canonical entropy obtained from the exact partition function (Ziff, Uhlenbeck, and Kac construction), and (iii) the entropy of a multinomial distribution with Boltzmann categorical probabilities and temperature determined from Clausius’ equation. It is well-known that the grand-canonical Bose–Einstein systematically overestimates entropy of canonical systems in regimes where particle-number fluctuations are significant. The exact canonical entropy correctly enforces the particle-number constraint, but recent experimental results suggest that it also overestimates particle-number fluctuations below the crossover temperature. The multinomial distribution is less common in thermodynamics. It addresses in a mathematically exact way the puzzle of the famous −log(N!) term introduced by Gibbs as a deus ex machina in discussions of thermodynamic entropy. One remarkable consequence is that the entropy of the multinomial distribution overcomes the issue of negative entropy at low temperature that affects the Gibbs and the Sackur–Tetrode entropies at low temperature. The analysis presented in the paper shows that the multinomial distribution, equipped with a categorical distribution calibrated in such a way that the resulting multinomial entropy fits Clausius’ equation, provides accurate approximations to the canonical entropy in the classical regime, while it is smaller than the canonical entropy below the crossover temperature. One feature of the multinomial distribution is that it predicts lower peak variance of the number of particles in the ground state than the canonical distribution. This is in agreement with recent experimental results; hence, this paper identifies the thermodynamically calibrated multinomial distribution as a candidate alternative to the canonical distribution for thermodynamic bosonic entropy in finite systems.

## 1. Introduction

The thermodynamic description of an ideal bosonic quantum gas depends on the statistical ensemble adopted. The grand-canonical formulation of Bose and Einstein [1,2] leads to a product of geometric distributions for the occupancy numbers. While analytically convenient, this formulation allows fluctuations in the total number of particles that are not present in the canonical ensemble, which is the appropriate ensemble when the gas is constrained inside a bounded region of space. In the thermodynamic limit, fluctuations of the particle number become relatively small, and canonical and grand-canonical ensembles become equivalent; however, for finite systems this equivalence is only approximate, and the difference between the two ensembles can become quantitatively relevant. In particular, the grand-canonical entropy systematically overestimates the canonical entropy in regimes where number fluctuations are significant, the discrepancy being especially relevant in finite systems and below the crossover temperature.

The canonical ensemble was analyzed in detail by Ziff, Uhlenbeck, and Kac (ZUK) [3], who worked out the exact canonical distribution of the occupancy numbers and clarified the structure of the canonical partition function for the ideal quantum gas. The canonical construction enforces the particle-number constraint exactly and therefore eliminates the unwanted fluctuations present in the grand-canonical ensemble.

However, the recent theoretical and experimental studies [4,5,6] on condensate fluctuations, i.e., the variance of the occupation number of the ground state, in finite Bose gases have highlighted that condensate fluctuations may also be significantly suppressed with respect to canonical predictions, revealing measurable differences between canonical, microcanonical, and related finite-particle-number descriptions in the vicinity of the Bose–Einstein condensation crossover temperature; see [4,5,6]. The sensitivity to the choice of ensemble has a long history in the theory of finite Bose systems. Early studies of condensate fluctuations in trapped Bose gases already emphasized the quantitative differences between competing finite-*N* descriptions and demonstrated that the crossover region remains particularly sensitive to the underlying statistical assumptions [7,8]. Subsequent investigations have continued to explore finite-size effects, condensate fluctuations, and alternative ensemble constructions, further supporting the view that the occupation statistics of finite Bose gases are not yet an experimentally settled issue [9,10]. This evidence motivates a critical re-examination of the canonical occupation statistics commonly employed in this regime and encourage the exploration of alternative finite-*N* occupation laws based on different physical preparation principles.

In this paper we explore the multinomial distribution, which is a direct consequence of the assumption of independent and identically distributed (i.i.d.) single-particle energy levels, by analyzing the entropies of the ideal Bose gas in the grand-canonical, canonical, and multinomial distributions across temperature ranges spanning the classical, crossover, and quantum-degenerate regimes. One of the merits of the multinomial distribution is that its entropy extends consistently beyond the classical regime. As recently shown in [11], this extension originates from the contribution of the denominator of the multinomial coefficient to the occupancy statistics. This contribution is absent in the original Gibbsian formulation and resolves the long-standing puzzle associated with the Gibbs correction term −log(N!), which continues to be the subject of investigation today [12,13,14].

The application of the multinomial distribution to equilibrium thermodynamics is obtained here by a thermodynamic calibration of the multinomial distribution, i.e., by deriving the temperature of heat bath and system from Clausius’ equation. Following this approach, the paper shows that, as T→∞, the multinomial entropy tends to the ZUK entropy, leading to a good agreement between the two in the classical regime. The numerical results presented here show that, below the crossover temperature, the condensate fluctuations predicted by the multinomial distribution are substantially smaller than those predicted by the canonical distribution. Since the experimental results reported in [4,5] show that the canonical ZUK distribution systematically overestimates fluctuations of finite-*N* systems, the numerical results presented in this paper motivate further investigation of multinomial occupation statistics as an alternative finite-*N* occupation law that preserves the thermodynamic one-particle population of energy levels while replacing equilibrium-induced Bose correlations by the i.i.d. assumption.

In this paper, the grand-canonical, canonical and multinomial statistical descriptions are presented in separate sections in order to emphasize that they are independent probabilistic constructions rather than variants of a single model. Their quantitative comparison is then carried out in Section 6.

## 2. Occupancy Numbers and Thermodynamics

Consider a system of *N* particles of the same species; let C={1,2,⋯,|C|}, be the set of quantum eigenstates allowed to a particle, let $c_{i}\in C,\;i=1,2,\cdots ,N,$ represent the generic eigenstate of the *i*-th particle, and let $H_{c}$ be the Hilbert space spanned by the set of eigenstates belonging to C. Also, let $s=(c_{1},c_{2},\cdots ,c_{N})$, $s\in S=C^{N}$, represent the generic eigenstate of the system in the full system Hilbert space $H_{s}=H_{c}^{⊗N}$ (the so-called microstate in many textbooks, e.g., [15]). The number of particles that occupy eigenstate *c* is

(1) $$\begin{array}{c} n_{c}\left(s\right)=\sum_{i=1}^{N}\delta_{c,c_{i}}, \end{array}$$

where

(2) $$\begin{array}{c} \delta_{x,y}=\left\{\begin{array}{cc} 1, & \mathrm{if}\;x=y,\\ 0, & \mathrm{elsewhere}. \end{array}\right. \end{array}$$

In the following, when unnecessary, we will omit the dependence of the occupancy number on *s* and we will denote *n*, $n=(n_{1},n_{2},\cdots ,n_{\left|C\right|}),$ the vector of the occupancy numbers, or, in short, the occupancy vector. The size |N| of the set N of the occupancy vectors allowed for a system with *N* particles is

(3) $$\begin{array}{c} \left|N\right|=\frac{(N+|C|-1)!}{N!(|C|-1)!}. \end{array}$$

Let the subset $S_{n}$ of S be the set of all vectors *s* that have the same vector n(s) of occupancy numbers. $S_{n}$ contains all and only the $W_{n}$ permutations of anyone of its vectors, where

(4) $$\begin{array}{c} W_{n}=\left\{\begin{array}{cc} \frac{N!}{\prod_{c\in C}n_{c}!}, & \mathrm{if}\;\sum_{c\in C}n_{c}=N,\\ 0, & \mathrm{elsewhere}, \end{array}\right. \end{array}$$

is the multinomial coefficient. When *n* spans the entire set N, the set $\left\{S_{n}\right\}$ forms a disjoint partition of S, ${⋃}_{n\in N}S_{n}=S,\;\;{⋂}_{n\in N}S_{n}=\emptyset$; hence each s∈S belongs to one and only one of the elements of $\left\{S_{n}\right\}$.

The physical Hilbert space of a system of bosons is the symmetric subspace $H_{n}$ of the full Hilbert space $H_{s}$; see [16] for a tutorial on symmetric Hilbert spaces. In the common case of additivity of the Hamiltonian, the expected system’s energy $\mu_{E}$ (the symbol μ is extensively used throughout the paper to indicate a mean value, and should not be confused with the chemical potential) is obtained from the probability distribution of the occupancy numbers $\left\{P_{n}\right\}=\left\{P_{n_{1}},P_{n_{2}},\cdots ,P_{n_{\left|C\right|}}\right\}$ as

(5) $$\mu_{E}=\sum_{n\in N}P_{n}E_{n},$$

where $E_{n}$ is the energy of the system with occupancy vector *n*, that is

(6) $$E_{n}=\sum_{c\in C}n_{c}\epsilon_{c},$$

$\left\{\epsilon_{c}\right\}$ being the set of the energy eigenvalues of the one-particle eigenstates. In the case of a non-relativistic monoatomic particle confined inside a D-dimensional box with all sides of length *L*, the energy eigenvalues expressed in *k*-units with aperiodic boundary conditions are

(7) $$\epsilon_{i_{1},i_{2},\cdots ,i_{D}}=\sum_{d=1}^{D}\frac{h^{2}i_{d}^{2}}{8kmL^{2}},\;\;i_{d}=1,2,\cdots ,\;\;d=1,2,\cdots ,D,\;\;c=\left(i_{1},i_{2},\cdots ,i_{D}\right),\;$$

where *m* is the mass of the particle, $k=1.38\times 10^{-23}\;\;J/K$ is Boltzmann’s constant and $h=6.626\times 10^{-34}\;J\cdot s$ is Planck’s constant. For large values of expected energy per particle, the discrete spectrum of energy eigenvalues can be approximated to a continuous one by introducing the so-called density of states. With low expected energy, the quality of the approximation can be improved by separating the ground state from the other states and treating the other states as dense; see [17]. However, although the density of states can speed up calculations, we prefer to avoid non-necessary approximations; therefore, in the following, we will consider the discrete spectrum of energy eigenvalues (7).

When the state of the bosonic system is mixed and the density matrix of the system is expressed in its own eigenbasis, the density matrix is diagonal and the von Neumann entropy $H_{n}$ of the bosonic system is equal to the Shannon entropy of the distribution $\left\{P_{n}\right\}$, that is

(8) $$H_{n}=-\sum_{n\in N}P_{n}\log\left(P_{n}\right),$$

where log(·) is the natural logarithm. For convenience, in (6)–(8) we express entropy and energy in Boltzmann’s units, called *k*-units for brevity in the following, so $H_{n}$ is non-dimensional and $\mu_{E}$ has the dimension of Kelvin degrees. The definition of entropy (8) applies to a system at the thermal equilibrium with a heat bath. In this context, all thermodynamic properties of the system derive from Clausius’ definition of temperature, which applies when an infinitesimally small reversible transformation at constant density (number of particles per unit volume) is considered:

(9) $$\begin{array}{c} \frac{dH_{n}}{d\mu_{E}}=\frac{1}{T}, \end{array}$$

where *T* is the temperature, expressed in Kelvin degrees, of the heat bath and system in equilibrium with it. When the entropy of the occupancy numbers is such that the temperature coincides with (9), many textbooks of thermodynamics and statistical mechanics call $S=kH_{n}$ thermodynamic entropy and $U=k\mu_{E}$ internal energy. In the case of *N* particles in a three-dimensional cubic box, the Bose–Einstein condensation (BEC) temperature $T_{BEC}$ is

(10) $$\begin{array}{c} T_{BEC}=\frac{h^{2}N^{2/3}}{\zeta {\left(3/2\right)}^{2/3}2\pi mkL^{2}}\approx 0.527\cdot \frac{h^{2}N^{2/3}}{2\pi mkL^{2}}, \end{array}$$

where ζ(·) is the Riemann zeta function.

## 3. Grand-Canonical Occupancy Statistics

The celebrated analysis by Bose and Einstein applies to an open system that randomly exchanges indistinguishable particles (bosons) with the surrounding environment in thermal equilibrium with it. The random nature of the exchange makes the total number of particles of the system a random variable. As mentioned in the introduction, when the expected number of particles of the system is large, the random fluctuations of the particle number around its mean value are relatively small, and the grand-canonical model can closely approximate the canonical one.

The derivation of Bose and Einstein was based on the semi-classical approach where quantum states are modelled as cells of volume $h^{D}$ in phase space. Here, to adapt the model of an open system to that of a closed system, we adopt the modern approach based on the energy eigenvalues obtained from the boundary conditions imposed by the container enclosing the system, see, e.g., [18]. It is a standard result that, with the system’s expected number of particles $\mu_{N}$ and expected energy $\mu_{E}$ as constraints, the entropy-maximizing distribution is the product of geometric distributions, one for each quantum eigenstate:

(11) $$P_{n}=\prod_{c\in C}\left(1-e^{-\beta \epsilon_{c}-\alpha }\right)e^{-n_{c}\left(\beta \epsilon_{c}+\alpha \right)}.$$

The expected number of particles in quantum eigenstate *c* is the celebrated BE (Bose–Einstein) statistics:

(12) $$\begin{array}{c} \mu_{c}=\frac{1}{e^{\beta \epsilon_{c}+\alpha }-1}. \end{array}$$

The two Lagrange multipliers β and α that impose the constraints in constrained entropy maximization are found by solving the system made by the two equations

(13) $$\mu_{N}=\sum_{c\in C}\mu_{c},$$

(14) $$\mu_{E}=\sum_{c\in C}\mu_{c}\epsilon_{c}.$$

The entropy of the occupancy numbers is

(15) $$\begin{array}{c} H_{n}=\alpha \mu_{N}+\beta \mu_{E}-\sum_{c\in C}\log\left(1-e^{-\beta \epsilon_{c}-\alpha }\right). \end{array}$$

After the imposition of Clausius’ equation, the Lagrange multiplier β turns out to be equal to $T^{-1}$ and −αkT is what today we call the chemical potential. (Note that throughout this paper we have β=1/T, not the common β=1/kT, because we express energy in Boltzmann units; therefore the Boltzmann constant is incorporated inside the denominator of the energy eigenvalue $\epsilon_{c}$.) Einstein shows in [2] that, in the classical regime, energy and entropy become equal to

(16) $$\mu_{E}\approx \mu_{N}\frac{DT}{2},$$

(17) $$H_{n}\approx \mu_{N}\log\left(\frac{eL^{D}{\left(2\pi emkT\right)}^{D/2}}{\mu_{N}h^{D}}\right).$$

In the thermodynamic limit, $\mu_{N}$ can be substituted by *N* and the last formula becomes the semi-classical entropy formula (here expressed in *k*-units) originally derived by Sackur and Tetrode; see, e.g., [15].

## 4. Canonical Occupancy Statistics

It is well-known that the canonical occupancy distribution can be derived by conditioning the grand-canonical BE occupancy distribution on a fixed total number of particles. In the information-theoretic approach, the canonical distribution is obtained from the constrained optimization of the entropy of a system with a fixed number *N* of bosons with the constraints on the system’s energy and

(18) $$\sum_{n\in N}P_{n}=1.$$

With both approaches, the result is the probability distribution found in [3], which is

(19) $$P_{n}=\frac{\prod_{c\in C}e^{-\beta \epsilon_{c}n_{c}}}{Z_{N}},$$

where $Z_{N}$ is the *N*-particle partition function and the Lagrange multiplier β is found by imposing the energy constraint. The entropy of the canonical system is

(20) $$\begin{array}{c} H_{n}=\beta \mu_{E}+\log\left(Z_{N}\right). \end{array}$$

Again, imposing Clausius’ equation one gets $\beta =T^{-1}$.

The partition function can be efficiently computed by the recursion of Borrmann and Franke [19]:

(21) $$Z_{n}=\frac{1}{n}\sum_{l=1}^{n}Z_{n-l}Q_{l},\;n=1,2,\cdots ,N,$$

where $Z_{0}=1$, $Q_{l}=\sum_{c\in C}e^{-\beta l\epsilon_{c}},\;\;l=1,2,\cdots ,N.$ The dominant cost of the recursion scales as $O(N^{2}+|C|N).$ With the refinement of the computational strategy proposed in [20], the computational cost scales to O(|C|N) once the single-particle partition sums $Q_{l}$ are known.

A useful interpretation of the canonical partition function is obtained through the cycle decomposition, where the quantity $Q_{l}$ is interpreted as the statistical weight of a collective group of *l* particles. Iterating the recursion leads to the expansion

(22) $$\begin{array}{c} Z_{N}=\sum_{\left\{m_{l}\right\}}\prod_{l=1}^{N}\frac{Q_{l}^{m_{l}}}{m_{l}!\;l^{m_{l}}}, \end{array}$$

where the sum runs over all non-negative integers ${\left\{m_{l}\right\}}_{l=1}^{N}$ satisfying $\sum_{l=1}^{N}l\;m_{l}=N.$ The integer $m_{l}$ is the number of groups of *l* particles that are treated collectively rather than independently. In particular, $m_{1}$ is the number of particles treated independently. For example, $m_{1}=N$ corresponds to a situation in which all particles behave independently, while $m_{N}=1$ corresponds to a fully collective *N*-particle contribution. In this perspective, we see that the cycles with l>1 bring *l*-body correlations at the particle level into the picture.

In the classical regime, correlations between particles become negligible and the dominant contribution to the partition function comes from the term l=1. The partition function therefore reduces approximately to

(23) $$\begin{array}{c} Z_{N}\approx \frac{Q_{1}^{N}}{N!}=\frac{Z_{1}^{N}}{N!}. \end{array}$$

In this limit, particles behave independently at the single-particle level, and the Gibbs correction factor N! emerges automatically, leading to Gibbsian entropy

(24) $$\begin{array}{c} H_{n}\approx H_{s}-\log\left(N!\right)=NH_{c}-\log\left(N!\right) \end{array}$$

where $H_{s}$ is the Shannon entropy of distinguishable particles (what we call in this paper the entropy of microstates) and $H_{c}$ is the one-particle entropy, i.e., the Shannon entropy of the one-particle Boltzmann distribution:

(25) $$P_{c}=\frac{e^{-\beta \epsilon_{c}}}{Z_{1}},\;\;Z_{1}=\sum_{c\in C}e^{-\beta \epsilon_{c}}.$$

Looking at (24) we immediately recognize that $NH_{c}$ is the Shannon–Gibbs entropy of microstates and that −log(N!) is the Gibbsian correction term. Equation (24) can be taken as the starting point for the derivation of the Sackur–Tetrode entropy formula, even if, historically, Sackur and Tetrode based their findings on a semi-classical approach that treats quantum states as cells of volume $h^{D}$ in phase space. Specifically, by approximating the sum that defines $Z_{1}$ in (25) to an integral, one gets

(26) $$Z_{1}\approx {\left(\frac{2\pi mkL^{2}}{\beta h^{2}}\right)}^{D/2},$$

(27) $$\begin{array}{c} \mu_{E}\approx \frac{ND}{2\beta }, \end{array}$$

that, substituted in (24), leads to

(28) $$\;H_{n}\approx \frac{ND}{2}\left(\log\left(\frac{2\pi emkL^{2}}{\beta h^{2}}\right)\right)-\log\left(N!\right).$$

By substituting the first two terms of Stirling’s asymptotic (large-*N*) expansion

(29) $$\log\left(N!\right)=N\log\left(N\right)-N+0.5\log\left(2\pi N\right)+O\left(N^{-1}\right),$$

Equation (28) becomes the Sackur–Tetrode entropy formula.

## 5. Multinomial Occupancy Statistics

A system with independent single-particle states lives in the full Hilbert space $H_{s}$ and is characterized by the distribution $\left\{P_{s}\right\}$, which, by the assumption of independence, is

(30) $$P_{s}=\prod_{i=1}^{N}P_{c_{i}}.$$

However, system’s physical properties, among which is entropy, depend on the probability distribution of the occupancy numbers, or, in other words, on the projection of the system onto the symmetric Hilbert subspace $H_{n}$ of the full Hilbert space $H_{s}$. It is a standard result of probability theory that, with the further assumption of identical distribution $\left\{P_{c}\right\}$ of all *N* distributions $\left\{P_{c_{i}}\right\}$, the probability distribution of the occupancy numbers is the multinomial distribution:

(31) $$\begin{array}{c} P_{n}=W_{n}\prod_{c\in C}P_{c}^{n_{c}}; \end{array}$$

see also [21,22] for the multinomial distribution in the statistical-mechanics picture of thermodynamics. The multinomial distribution can be obtained by conditioning the product of Poisson distributions on a fixed *N* with mean values $\left\{\lambda_{c}\right\}$ such that

(32) $$\begin{array}{c} P_{c}=\frac{\lambda_{c}}{\sum_{c\in C}\lambda_{c}}. \end{array}$$

From this perspective, we recognize that the relation between the product of Poisson distributions and the multinomial distribution is analogous to the relation between the BE and ZUK distributions, where independent geometric occupancies of the grand-canonical BE ensemble become the ZUK distribution when conditioned on a fixed particle number.

In the context of probability theory, the one-particle probability distribution $\left\{P_{c}\right\}$ that equips the multinomial distribution is called the categorical distribution or generalized Bernoulli distribution, the trials being generalized (non-binary) Bernoulli (independent) trials and the |C| categories being the |C| one-particle eigenstates. Note the difference between the multinomial distribution and the BE and ZUK distributions: while here we start from the non-physical full Hilbert space and then project it onto its symmetric subspace, the BE and ZUK distributions directly analyze the system as an element of the symmetric Hilbert space.

The entropy of the multinomially distributed occupancy numbers is

(33) $$H_{n}=-E\{\log\left(P_{n}\right)\}\;$$

(34) $$\begin{array}{c} =-NE\left\{\log\left(P_{c}\right)\right\}-E\left\{\log\left(W_{n}\right)\right\}\; \end{array}$$

(35) $$\begin{array}{c} \;=NH_{c}-\log\left(N!\right)+\sum_{c\in C}E\left\{\log\left(n_{c}!\right)\right\}, \end{array}$$

where E{·} denotes the expectation with respect to the probability distribution of the random variable or random vector, whether $c,n_{c}$, or *n*, appearing inside the curly brackets. Specifically, since $n_{c}$ is a a binomial random variable $\left(N,P_{c}\right)$, we write

(36) $$E\{\log\left(n_{c}!\right)\}=\sum_{n_{c}=0}^{N}P_{n_{c}}\log\left(n_{c}!\right)\;$$

(37) $$\begin{array}{cc} \; & =\sum_{n_{c}=2}^{N}\left(\begin{array}{c} N\\ n_{c} \end{array}\right)P_{c}^{n_{c}}{\left(1-P_{c}\right)}^{N-n_{c}}\log\left(n_{c}!\right),\; \end{array}$$

where

(38) $$\begin{array}{c} \left(\begin{array}{c} N\\ n \end{array}\right)=\frac{N!}{(N-n)!n!} \end{array}$$

is the binomial coefficient. As N→∞, manipulating the logarithm of the multinomial coefficient by Stirling’s asymptotic expansion, one promptly realizes that the entropy per particle $H_{n}/N$ tends to zero. For this reason, here and in the rest of the paper only entropy is considered, not entropy per particle. Numerical evaluation of the sum (39) can be efficiently performed by applying to each binomial term the integral representation of [23], which expresses $E\{\log\left(n_{c}!\right)\}$ as a one-dimensional integral over (0,∞). The dominant cost per eigenstate is O(1), so the total cost of evaluating the sum scales as O(|C|) with the number of single-particle eigenstates retained. This should be contrasted with the recursive computation of the canonical partition function $Z_{N}$ via the Borrmann–Franke recursion (21), whose dominant cost scales as O(|C|N).

Compared with (24), which is an approximation to entropy rather than an exact Shannon entropy, the novelty introduced by (35) is the sum

(39) $$\begin{array}{cc} & \sum_{c\in C}E\left\{\log\left(n_{c}!\right)\right\}, \end{array}$$

that, when added to the other terms, makes $H_{n}$ a valid Shannon entropy and guarantees $H_{n}\geq 0$. Note that, thanks to the introduction of the denominator of the multinomial coefficient in the Gibbsian entropy Formula (24), the resulting entropy no longer suffers from the well-known pathology of (24) and the closely related Sackur–Tetrode entropy formula, which become negative at low temperatures just because of the absence of this term.

Before concluding this section, it is worth discussing the relationship between the multinomial approach and other approaches proposed in the past for the statistical description of systems with a fixed number of bosons. The multinomial distribution places both the Boltzmann multinomial counting factor and the Gibbs correction factor N!, contained in the numerator of the multinomial coefficient, within a unified exact probabilistic framework. Indeed, while the historical formulations of Boltzmann and Gibbs are not explicitly based on independent preparation, the multinomial counting factor coincides with the multiplicity generated by an i.i.d. occupancy model. It is instructive to regard the multinomial distribution as the collection of all multinomial coefficients $\left\{W_{n}\right\}$ compatible with *N*, each one weighted by the probability $\prod_{c\in C}P_{c}^{n_{c}}$ of any one of the equiprobable microstates whose occupancy number is *n*. From this perspective, we recognize the weaknesses of the Gibbsian entropy (24) and of the Boltzmannian entropy S=klog(W), where *W* is the multiplicity of microstates of the most probable occupancy vector. In S=klog(W), *W* has the meaning of the inverse of the probability of microstates of the most probable occupancy vector. Here, the weakness is that all other occupancy vectors are disregarded. The consequence is that the Boltzmannian approach holds only when the system can reasonably be represented by the most probable occupancy vector. This happens when all occupancy vectors concentrate around the most probable one, i.e., only in the thermodynamic limit. In the Gibbsian entropy, the probability of microstates with occupancy vector *n* is $\prod_{c\in C}P_{c}^{n_{c}}$ and all occupancy vectors are considered. The weakness is that their multiplicity $W_{n}$ is only partially taken into account, as only the numerator of $W_{n}$ appears in (24). As a consequence, the Gibbsian entropy Formula (24) holds only in the classical regime, where the probability that two or more bosons occupy the same quantum state is negligible. Due to these weaknesses, the well-known claim of equivalence between the Boltzmannian approach, possibly corrected in some appropriate way to take into account indistinguishability of bosons (this is actually another weakness of S=klog(W) that needs to be addressed), and the Gibbsian approach, holds only when the two hold simultaneously, i.e., in the thermodynamic limit and in the classical regime, while the multinomial distribution, removing all these weaknesses, acts as a bridge between the two, allowing the representation of systems with a small number of bosons in the non-classical regime. A remarkable difference between the ZUK distribution and the multinomial distribution is the following. In the ZUK distribution the probability of an occupancy vector is proportional to $\prod_{c\in C}P_{c}^{n_{c}}$. With standard weights of the exponential type, this means that the probability of an occupancy vector depends only on its energy, so two occupancy vectors with the same energy have the same probability, even if one has $10^{23}$ times the microscopic configurations of the other. From a combinatorial viewpoint, this differs from the traditional Boltzmann intuition that multiplicity should contribute directly to probability assignments, an intuition that is preserved by the multinomial distribution in which the probability of a microstate (not of an occupancy vector) is proportional to $\prod_{c\in C}P_{c}^{n_{c}\left(s\right)}$, while the probability of the occupancy vector is proportional to the probability of microstates multiplied by their multiplicity.

### Thermodynamically Calibrated Categorical Distribution of the Multinomial

By imposing the Boltzmann one-particle probability distribution (25) as the categorical distribution, we get the many-particle Boltzmann distribution for the probability distribution of microstates

(40) $$P_{s}=\frac{\prod_{i=1}^{N}e^{-\beta \epsilon_{c_{i}}}}{Z_{1}^{N}}=\frac{\prod_{c\in C}e^{-\beta \epsilon_{c}n_{c}\left(s\right)}}{\sum_{n\in N}W_{n}\prod_{c\in C}e^{-\beta n_{c}\epsilon_{c}}},$$

where we have substituted the multinomial expansion of $Z_{1}^{N}$,

(41) $$\begin{array}{c} Z_{1}^{N}={\left(\sum_{c\in C}e^{-\beta \epsilon_{c}}\right)}^{N}=\sum_{n\in N}W_{n}\prod_{c\in C}e^{-\beta n_{c}\epsilon_{c}}. \end{array}$$

For the probability distribution of the occupancy numbers we get the multinomial distribution in the form

(42) $$P_{n}=\frac{W_{n}\prod_{c\in C}e^{-\beta \epsilon_{c}n_{c}}}{\sum_{n\in N}W_{n}\prod_{c\in C}e^{-\beta n_{c}\epsilon_{c}}},$$

whose entropy is

(43) $$H_{n}=\beta \mu_{E}+N\log\left(Z_{1}\right)-\log\left(N!\right)+\sum_{c\in C}E\left\{\log\left(n_{c}!\right)\right\}.$$

By imposing Clausius’ equation, we find *β* from

(44) $$\begin{array}{c} \frac{dH_{n}}{d\mu_{E}}=\beta +\frac{\sum_{c\in C}dE\left\{\log\left(n_{c}!\right)\right\}}{d\mu_{E}}=\frac{1}{T}. \end{array}$$

In the classical regime, where both the sum (39) and its derivative w.r.t. system’s energy are negligible, we arrive again to $\beta =T^{-1}$ and to the Sackur–Tetrode entropy Formula (17). Note however that, below the crossover temperature, the possibly strong dependence of the sum on $\mu_{E}$ can make *β* substantially different from the inverse temperature. Also note that the derivative of the sum needs to be approximated numerically.

## 6. Numerical Results

This section presents numerical results obtained for systems with 10 and 100 particles of mass $1.6735575\times 10^{-27}$ kg in a cubic 3D box with a side length of $10^{-7}$ meters. The number of quantum states is truncated to $33^{3}$. The BEC temperatures are $7.4\times 10^{-4}$ and $3.4\times 10^{-3}$ Kelvin degrees for N=10 and N=100, respectively. In the Bose–Einstein case, the number of particles is intended to be the mean particle number.

**Figure 1:**
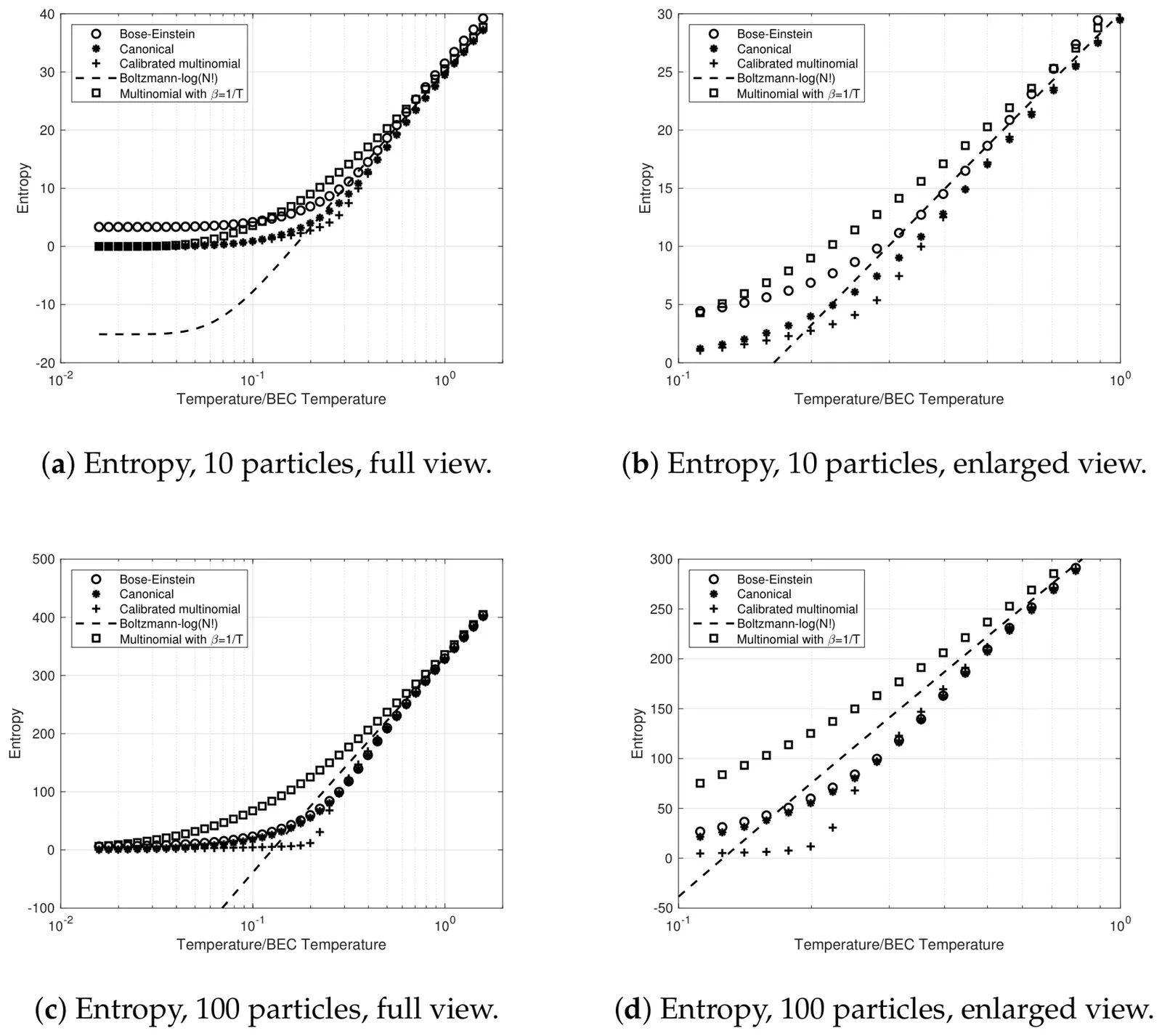
Various entropies in *k* units versus temperature for systems of 10 and 100 particles in three dimensions. The circles denote the Bose–Einstein entropy, the asterisks the canonical (ZUK) entropy, the plus symbols the thermodynamically calibrated multinomial entropy, the squares the multinomial entropy with β=1/T, and the dashed line the Gibbsian approximation (24).

Figure 1 reports entropy in various cases. As expected, at low temperatures, the Bose–Einstein entropy is substantially greater than the ZUK entropy and that of the thermodynamically calibrated multinomial distribution. Actually, all particles tend to occupy the few quantum states with low energy, and, in the limit of zero temperature, only the ground state. However, the randomness in the particle number is such that the system’s entropy tends as T→0 to the entropy of a geometrically distributed random variable with mean $\mu_{N}$. This effect is particularly pronounced for 10 particles, for which fluctuations in the particle number around the mean value is relatively large. Accordingly, the entropy of the ZUK distribution remains well below the entropy of the Bose–Einstein distribution, which is dominated by the mentioned fluctuations. As expected, the difference between the entropy of the ZUK distribution and that of the multinomial distribution becomes negligible in the classical regime, while the two enlarged views clearly show that the difference is non-negligible below the BEC temperature. Note that the multinomial distribution is obtained by restricting the set of allowed distributions to the family of multinomial distributions, so it is not surprising that the resulting entropy is always lower than the entropy of the ZUK distribution, whose search space is the entire space of all legal occupancy distributions. We further observe that, both with 10 and with 100 particles, the multinomial distribution with the formal substitution β=1/T has an entropy greater than that of the other models over a broad range of temperatures, but this distribution does not satisfy Clausius’ equation; hence, we can say that, in this case, the concept of temperature itself does not apply. The Gibbsian approximation to entropy (24) surpasses the canonical entropy over a range of temperatures that is clearly visible in the enlarged views, especially in the case of 100 particles. However, (24) is a high-temperature approximation to entropy; it is not the entropy itself. As discussed before, the canonical entropy differs from the Gibbsian entropy for the presence of cycles of length greater than one. Preliminary analysis of the two-particle canonical partition function already indicates that the contribution of the second-order cycle to the entropy at fixed inverse temperature can be negative, in which case the ZUK entropy is lower than the Gibbs entropy. However, further investigation of higher-order cycles is beyond the scope of the present paper.

**Figure 2:**
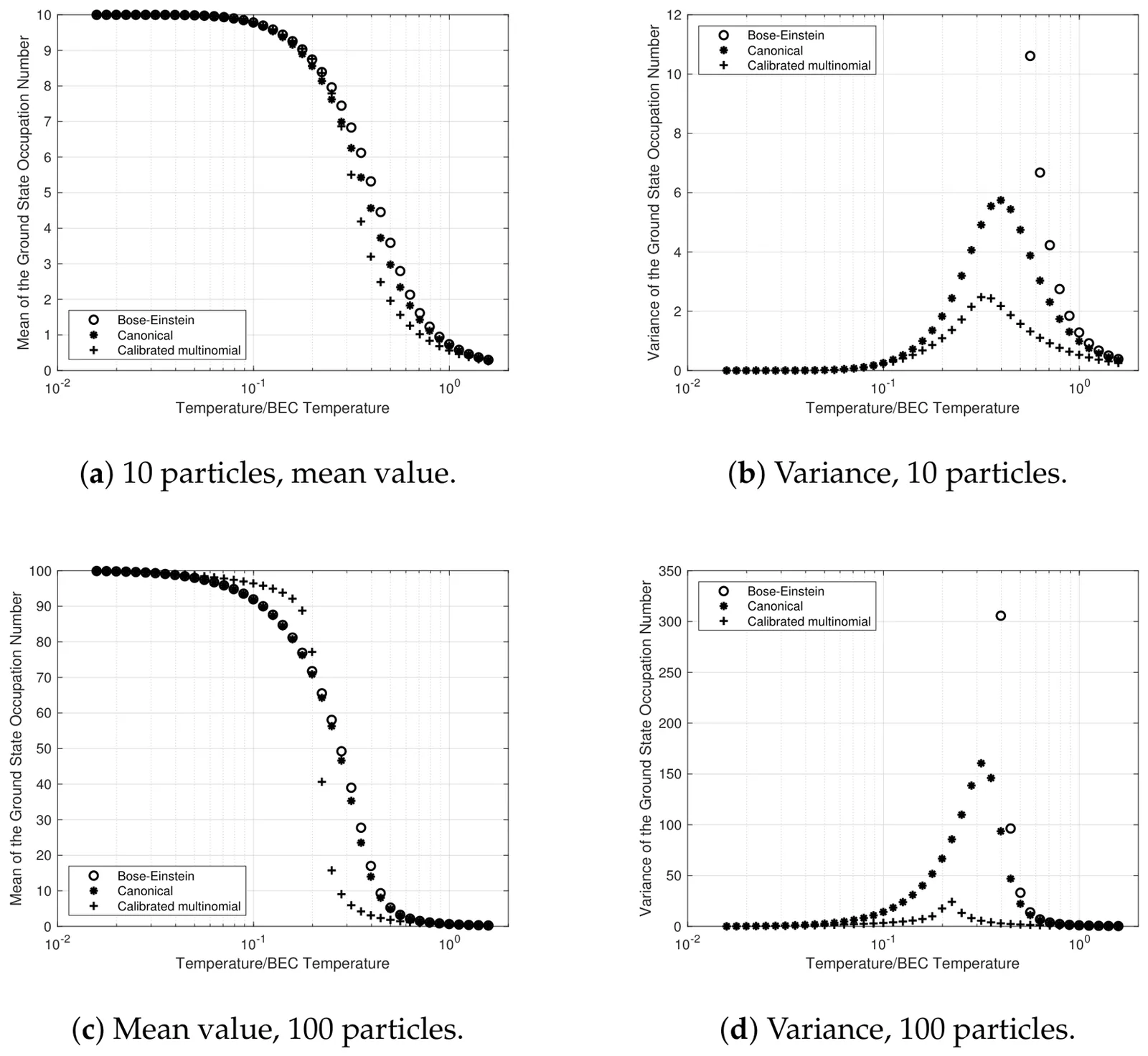
Mean value and variance of the occupation number of the ground state versus temperature for systems of 10 and 100 particles. The circles are the Bose–Einstein, the asterisks are the the canonical (ZUK) distribution, the plus symbols are the thermodynamically calibrated multinomial distribution.

Figure 2 reports the expected value and variance of the occupancy number of the ground state for 10 and 100 particles. The peak variance predicted by the multinomial model is N/4, obtained from the variance formula NP(1−P) with P=0.5. The ratio between the peak variance of the ZUK distribution and that of the multinomial distribution is 2.3 for 10 particles, 6.4 for 100 particles, while our calculations, not presented here, yield 16 for 1000 particles. In this regard, we observe that reference [4] reports that the peak variance experimentally follows the law $N^{1.134}$, but the peak variance of the ZUK with the numbers of particles considered here does not seem to follow a law of the form $N^{x}$.

## 7. Conclusions

In this work, we have compared three probability distributions for a finite ideal bosonic gas: the grand-canonical Bose–Einstein distribution, the exact canonical distribution of Ziff, Uhlenbeck, and Kac, and the multinomial distribution based on the Boltzmann categorical distribution with a *β* such that its entropy satisfies Clausius’ equation. We have presented numerical results for a system of 10 particles and for a system of 100 particles in a three-dimensional box. The numerical results confirm that the grand-canonical Bose–Einstein entropy systematically overestimates the canonical entropy in finite systems, the discrepancy being most pronounced at low temperatures and for small particle numbers, where particle-number fluctuations are relatively large. For the 10-particle system, the gap between the Bose–Einstein entropy and the canonical entropy is substantial at low-to-intermediate temperatures. For the 100-particle system, the gap is reduced but remains clearly visible at low temperatures, as shown in Figure 1d. Recent results from [4,5] show that the measured fluctuations of the ground state below the BEC temperature are more suppressed than those predicted by the canonical distribution. The numerical results reported in Figure 2b,d show that the peak fluctuations of the ground state associated with our thermodynamically calibrated multinomial distribution are below those predicted by the ZUK distribution. Therefore the findings of this paper motivate future investigation of the thermodynamically calibrated multinomial distribution as an alternative to the canonical distribution.

## Data Availability

The original contributions presented in this study are included in the article. Further inquiries can be directed to the corresponding author.
